# Manufacturing, Properties, and Applications of Porous Ti_2_AlC: A Review

**DOI:** 10.3390/ma19102113

**Published:** 2026-05-18

**Authors:** Marek Potoczek

**Affiliations:** Department of Chemical Technology and Materials Science, Faculty of Chemistry, Rzeszow University of Technology, al. Powstańców Warszawy 12, 35-959 Rzeszów, Poland; potoczek@prz.edu.pl

**Keywords:** lightweight materials, porous MAX phase (Ti_2_AlC), emerging materials, nanolaminated structures, mechanical properties, physicochemical properties, applications

## Abstract

Porous Ti_2_AlC, a member of the MAX phase family of nanolaminated ternary carbides and nitrides, has attracted increasing attention due to its unique combination of metallic and ceramic properties. This review summarizes recent advances in the fabrication, structure–property relationships, and applications of porous Ti_2_AlC. Various processing routes, including incomplete sintering, sacrificial templating, replica techniques, gel casting, extrusion, and direct ink writing, are compared in terms of achievable porosity, pore morphology, and structural control. Particular emphasis is placed on the role of porosity in tailoring mechanical performance, thermal conductivity, and high-temperature oxidation resistance, based on available literature data. Recent progress in applications of porous Ti_2_AlC and related MAX phases is also discussed, including their use in filtration, membrane supports, heat exchangers, electrochemical systems for hydrogen evolution, and as preforms for lightweight interpenetrating metal/MAX phase composites. Finally, current challenges and future research directions are identified, highlighting the need for improved control of porosity and a deeper understanding of structure–property relationships.

## 1. Introduction

Ti_2_AlC represents one of the most extensively investigated compounds within the MAX phase family. MAX phases are characterized as thermodynamically stable ternary carbides or nitrides possessing a nanolaminate crystal structure [1,2]. Scientific interest in these phases intensified during the late 20th century, following the discovery of the exceptional properties of Ti_3_SiC_2_ by Barsoum and El-Raghy [3]. These compounds are represented by the formula M_n+1_AX_n_, where M denotes an early transition metal, A signifies an element from groups 13–16 of the periodic table, X corresponds to carbon and/or nitrogen, and n = 1–3 [4]. The structural arrangement, combined with the coexistence of ionic, covalent, and metallic bonding, enables MAX phases to exhibit a unique combination of ceramic and metallic attributes. Dense Ti_2_AlC demonstrates a high compressive strength (540 MPa), flexural strength (275 MPa), Young’s modulus (277.6 GPa), shear modulus (118.6 GPa), and fracture toughness (6.5 MPa·m^1/2^) [5,6,7,8]. Furthermore, beyond its superior mechanical performance, Ti_2_AlC exhibits exceptional resistance to wet and high-temperature corrosion, radiation damage, and high-temperature self-healing capabilities [9,10,11,12,13].

Although numerous publications on dense Ti_2_AlC exist in the literature, the number of studies on porous Ti_2_AlC remains limited. To date, according to Scopus, there are 1999 records on “Ti_2_AlC”; however, there are only 49 on “porous Ti_2_AlC”.

A comprehensive overview of the synthesis routes and properties of dense MAX phases is provided in the book by Barsoum entitled “MAX Phases: Properties of Ternary Carbides and Nitrides”, published in 2013 [4]. In addition, numerous high-quality review articles addressing dense MAX phases are available in the literature [14,15,16,17,18]. In contrast, despite increasing research activity, porous MAX phases have received far less systematic attention. To date, only one limited review on porous MAX phases exists, in the form of a short chapter in the publication entitled “Processing of MAX phases: From synthesis to applications” [18].

The introduction of porosity significantly alters the physical, mechanical, and functional properties of materials, often over several orders of magnitude [19,20,21,22,23]. By controlling the total porosity, pore size, pore shape, and interconnectivity, it is possible to tailor material properties to specific application requirements. Porous ceramics are characterized by unique features such as low density, high permeability to fluids and gases, and enhanced surface area while retaining high-temperature stability and chemical resistance. Depending on the fabrication method, porous ceramics can be produced with porosities ranging from a few to more than 95 vol.% and with pore sizes ranging from the nanometer to millimeter scale. In addition to total porosity, key microstructural parameters governing the properties of porous ceramics include pore size distribution, pore morphology, connectivity, and the thickness of the solid struts separating adjacent pores. Moreover, porous Ti_2_AlC is a lightweight material. For a total porosity ranging from 10 to 90 vol.%, the density of the material varies between 0.41 and 3.70 g/cm^3^, assuming a theoretical density of Ti_2_AlC of 4.11 g/cm^3^ [18].

In ceramic materials, the introduction of porosity opens new opportunities for applications that are difficult or impossible to realize with dense materials. These include catalyst supports, filters, electrodes for harsh environments, heat exchangers, preforms for interpenetrating phase composites, and many others [21,22,23]. However, porosity reduces mechanical strength, modifies electrical and thermal properties, and may adversely affect oxidation resistance.

Therefore, the aim of this paper is to provide a comprehensive and critical overview of porous Ti_2_AlC with a particular focus on manufacturing routes, porosity–property relationships, and application potential. The effect of porosity on mechanical, thermal, and oxidation behavior is systematically discussed based on the available literature data. Potential applications that are unattainable in dense materials are also presented. Furthermore, current challenges and future research directions for porous Ti_2_AlC are highlighted.

## 2. Manufacturing of Porous Ti_2_AlC

**Figure 1:**
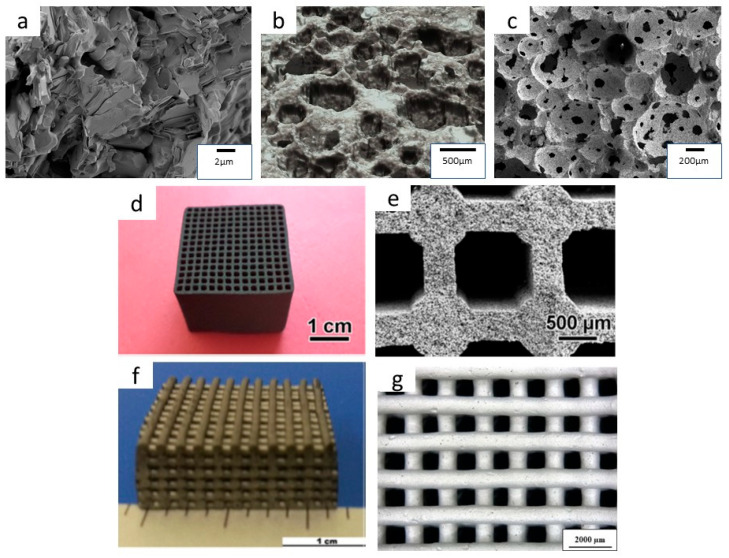
Examples of porous Ti_2_AlC typically obtained for various manufacturing routes: (**a**) incomplete sintering (unpublished author’s image), (**b**) sacrificial template (unpublished author’s image), (**c**) gel casting of foams [37] (adapted with permission from [37]; copyright 2018 John Wiley and Sons), (**d**,**e**) extrusion of honeycombs [38] (adapted with permission from [38]; copyright 2015 John Wiley and Sons), (**f**,**g**) direct ink writing [39] (adapted with permission from [39]; copyright 2019 Elsevier).

The pore architecture of porous ceramics strongly depends on the fabrication method [21,22,23,24,25,26]. Various processing techniques have been employed to produce porous Ti_2_AlC, including incomplete densification or reaction sintering of elemental powders, sacrificial templating, replica method, gel casting of foams, extrusion, and direct ink writing (DIW) [27,28,29,30,31,32,33,34,35,36,37,38,39]. Table 1 summarizes the porous Ti_2_AlC fabrication methods reported in the literature, together with the corresponding achievable ranges of total porosity and pore size. Figure 1 illustrates the pore architectures typically obtained for different manufacturing routes.

### 2.1. Incomplete Sintering

The incomplete sintering method leads to the formation of materials with relatively low porosity, typically up to several volume percent. In this approach, densification is deliberately limited by reducing the sintering temperature and/or shortening the sintering time. Although this method is simple and does not require additional processing steps, it offers limited control over pore volume fraction, size, and morphology [27].

### 2.2. Sacrificial Template Method

The sacrificial template method involves mixing Ti_2_AlC powders with a temporary space holder material of well-defined particle size [28,29,30,31,32,33]. The resulting powder mixture is shaped, typically by uniaxial or isostatic pressing, followed by removal of the space holder through dissolution (e.g., NaCl, saccharose) or thermal decomposition (e.g., NH_4_HCO_3_). Subsequently, the porous body is sintered under pressureless conditions. The total porosity is controlled by the amount of added space holder, while the pore size depends on the grain size of the space holder.

### 2.3. Replica Method

The replica method is based on the infiltration of a ceramic slurry into a porous polymeric template, most commonly a polyurethane foam [34]. After impregnation, excess slurry is removed, and the coated template is dried. During subsequent thermal treatment, the polymeric substrate is burned out, leaving behind a ceramic structure that replicates the morphology of the original foam. Final densification is achieved by sintering. This technique enables the fabrication of highly porous ceramic foams with porosities often exceeding 95 vol.% and pore sizes reaching even several millimeters. The pore size and architecture can be controlled by selecting polymer templates with different pore densities, typically expressed in pores per inch (ppi). Bowen and Thomas fabricated Ti_2_AlC foams using this method, employing a polyurethane sponge with a pore density of 10 ppi [34]. The resulting material exhibited a compressive strength ranging from 0.2 to 6.3 MPa, depending on the additional coating stages.

### 2.4. Gel Casting of Foams

The gel casting of foams is a versatile technique for producing porous ceramics with total porosity predominantly in the range of 50–95 vol.%. The process involves the preparation of a stable ceramic suspension containing ceramic powder, water, and dispersants. Foaming agents and gelling agents are then added, and the suspension is mechanically foamed. Foaming agents reduce the surface tension at the liquid–gas interface, facilitating bubble formation, while gelling agents stabilize the foam structure by forming a rigid hydrogel network in the liquid phase. Subsequent processing steps include drying, burnout of organic components, and high-temperature sintering. The total porosity is dependent on the foaming yield. Using agarose as a gelling agent, Ti_2_AlC foams with total porosities ranging from 54 to 93 vol.% and cell sizes between 20 and 615 µm have been produced [35,36,37].

### 2.5. Extrusion of Honeycomb Structures

Extrusion is commonly used to fabricate ceramic honeycomb structures with highly regular channel geometries. In this process, ceramic powders are mixed with solvents, binders, and plasticizers to obtain a plastic feedstock with suitable rheological properties. The feedstock is then extruded through a precision die to form a honeycomb structure, followed by drying and sintering. Using this technique, Fang et al. fabricated a Ti_2_AlC honeycomb monolith with channel sizes of approximately 1000 µm and microporosity within the strut in the range of 2–15 µm [38]. Such structures are particularly attractive for applications requiring high permeability, low pressure drop, and good thermal and electrical conductivity.

### 2.6. Direct Ink Writing

Direct ink writing (DIW) is an additive manufacturing technique that enables the fabrication of complex three-dimensional ceramic architectures based on digitally designed models [26]. In DIW, a viscoelastic ceramic ink containing ceramic powder and organic binders is extruded through a nozzle and deposited layer by layer to form a predefined structure. After printing, the green body is dried, organic additives are removed, and the structure is sintered. DIW offers exceptional control over macroscopic geometry, pore architecture, and spatial distribution of material, making it particularly suitable for customized components and biomedical applications. Elsayed et al. fabricated Ti_2_AlC lattices with total porosities ranging from 44 to 63 vol.% using DIW, where the macropore size was defined by the spacing between printed filaments [39].

### 2.7. Comparison of Fabrication Methods for Porous Ti_2_AlC

In the incomplete sintering method, the ratio of open porosity to total porosity is controlled by the selection of the initial powder particle size, pressing pressure, and sintering time and temperature. In contrast, a common feature of the other methods listed in Table 1 for the fabrication of ceramics with higher porosity is the pronounced predominance of open porosity. The small amount of closed porosity results from incomplete densification of the struts during sintering.

The choice of fabrication method for porous Ti_2_AlC depends on its intended application. The key parameters include open and total porosity, pore size and morphology, and pore interconnectivity. When small pore sizes are required, incomplete sintering or the sacrificial template method employing fine space holders should be selected [18]. In certain applications, a hierarchical pore structure combining both large and small pores is desirable. For instance, diesel particulate filters (DPFs) with a honeycomb structure contain millimeter-scale channels that facilitate the inflow of exhaust gases, while micropores within the channel walls enable the efficient capture of soot particles [38]. For materials with large pores, four principal fabrication methods can be employed: the sacrificial template method using large space holders, replication of porous foams, gel casting of foams, and additive manufacturing techniques such as 3D printing [23]. At the industrial scale, porous ceramic materials are most commonly produced via replication of porous polyurethane foams. This is primarily due to the widespread availability of such foams with well-defined pore sizes, typically specified in pores per inch (ppi), which allows straightforward control of pore size during processing. Additionally, shaping is relatively straightforward, as the desired geometry can be cut from a bulk foam block using a heated wire and subsequently impregnated with a ceramic slurry. Another approach to producing highly porous materials is gel casting of foams. Total porosity is controlled by the volume fraction of the generated foam, whereas pore size is governed by the gelation kinetics and foam stability, which can be adjusted through the use of surfactants. Due to the inherently unstable nature of foams, materials produced by this technique typically exhibit a broad pore size distribution. Gel casting belongs to the class of near-net-shape methods; thus, complex geometries are defined by the mold, and any final machining is usually limited to minor grinding operations [23]. Regular and well-defined macroporous structures can be achieved using direct ink writing (DIW), where the final geometry is determined by a computer-aided design (CAD) model. In the case of ceramic materials, precise control of the rheological properties of the printing paste is essential. The paste has to behave as a viscous fluid during extrusion through the nozzle, while immediately after deposition, it should exhibit solid-like behavior to prevent deformation during drying and sintering [39].

In terms of scalability, incomplete sintering is considered highly scalable, as it closely resembles conventional processing routes for dense ceramics and can be readily implemented at the industrial level. The process is primarily controlled by the particle size distribution of the starting powder, as well as by sintering temperature and time. Its main limitation, however, is the tendency to produce a non-uniform pore microstructure. Foam-based methods also exhibit high scalability, as production can be easily increased by employing larger mixing systems. Similarly, the sacrificial template method offers high scalability, since pore size and morphology can be tailored through the appropriate selection of the space holder. A comparable level of scalability is observed for polyurethane foam replication, although it relies on effective collaboration with polyurethane foam manufacturers. In contrast, additive manufacturing techniques such as direct ink writing exhibit lower scalability due to high equipment costs and relatively slow production rates. Nevertheless, these methods are among the most rapidly evolving shaping techniques for both dense and porous ceramics. These techniques are particularly well-suited for the fabrication of small, complex geometries and are especially promising in applications such as biomaterials and porous structures for electronics. For example, in biomedical applications, bone defect reconstruction can be achieved by first determining the precise defect geometry using computed tomography, followed by the development of a CAD model, which enables the fabrication of a patient-specific implant.

It should be noted that the machining of complex shapes from dense advanced ceramics is typically associated with high processing costs due to their high hardness (e.g., Al_2_O_3_, ZrO_2_, SiC, and Si_3_N_4_). However, this limitation does not apply to Ti_2_AlC. Dense Ti_2_AlC exhibits significantly lower hardness (2.8–4.5 GPa, Vickers hardness) compared to conventional advanced ceramics (e.g., 15–25 GPa for Al_2_O_3_) [4]. Consequently, one of the key advantages of Ti_2_AlC and other MAX phases is their excellent machinability. It has been demonstrated that dense Ti_2_AlC can be readily machined even using conventional machining tools such as a standard lathe [4,16]. Furthermore, due to its lower hardness, machining of porous ceramic materials is generally less demanding than that of their dense counterparts. From an economic perspective, the primary limitation in the production of both dense and porous Ti_2_AlC is not machining, but rather the high cost of the raw material (Ti_2_AlC powder), which is approximately 500 USD/kg [16].

## 3. Mechanical and Physicochemical Properties of Porous Ti_2_AlC

### 3.1. Compressive Strength

**Figure 2:**
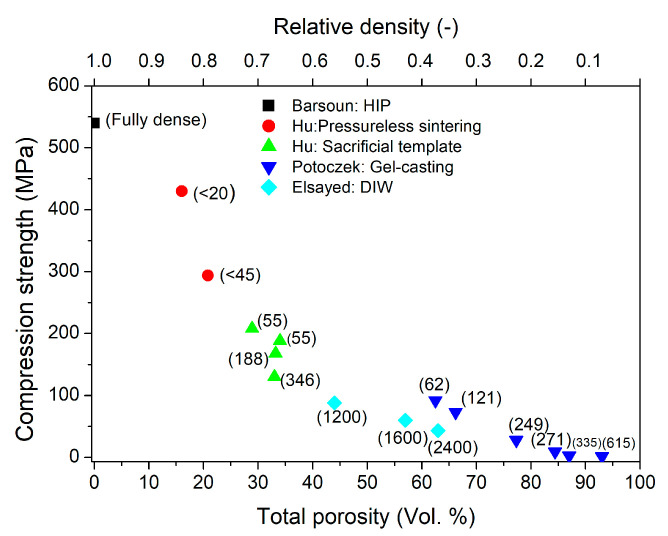
Reported compressive strengths of porous Ti_2_AlC as a function of total porosity. Numbers in parentheses indicate the average pore size [6,28,35,36,37,39].

The compressive strength of porous ceramics decreases with increasing porosity due to the increasing volume fraction of voids, which act as stress concentrators. Figure 2 presents the reported compressive strengths of porous Ti_2_AlC as a function of total porosity. The application of various forming techniques, including incomplete sintering, sacrificial templates, gel casting of foams, and direct ink writing (DIW), has enabled the fabrication of porous Ti_2_AlC with porosities ranging from 16 to 93 vol.% and compressive strengths varying from approximately 430 to 1.6 MPa [28,35,36,37,39]. For comparison, the compressive strength of fully dense Ti_2_AlC (540 ± 21 MPa) reported by Barsoun et al. [6] is also included in Figure 2. At a given porosity level, the compressive strength depends on pore size. Hu et al. demonstrated that for samples manufactured by the sacrificial template method with similar total porosity (29–33 vol.%), the compressive strength decreased from 208 to 130 MPa as the average pore size increased from 55 to 346 µm [28]. A similar trend is observed for samples fabricated using different processing techniques. As shown in Figure 2, specimens produced by the DIW method exhibit lower compressive strength than those obtained by the gel casting of foam technique. DIW-fabricated samples with a porosity of 63% are characterized by a pore size of approximately 2400 µm, whereas samples with the same porosity produced by gel casting of foams show a significantly smaller average pore size of about 110 µm (Table 1). An additional contributing factor is the difference in pore architecture between DIW and gel casting (Figure 1).

**Figure 3:**
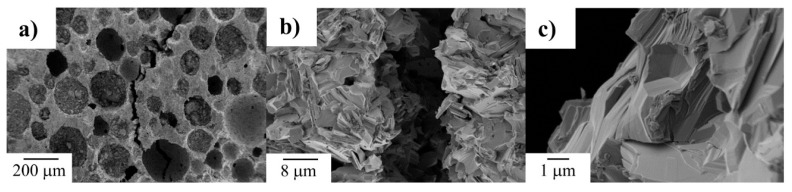
SEM images at different magnifications of the fracture surface of Ti_2_AlC after the compression test, with a total porosity of 66.2 vol% [37] (adapted with permission from [37]; copyright 2018 John Wiley and Sons). Magnifications: (**a**) 200x; (**b**) 5000x; (**c**) 25,000x;.

Typical deformation mechanisms in Ti_2_AlC and other MAX phases include kink band formation and delamination. These mechanisms dissipate a significant amount of mechanical energy during crack propagation, resulting in excellent damage tolerance [5,6]. Figure 3 presents SEM observations of porous Ti_2_AlC with a total porosity of 66.2 vol.% after compression testing, which revealed the formation of kink bands and delamination, although these were less pronounced than in dense Ti_2_AlC [37]. It should also be noted that the literature still lacks information on other important mechanical properties of porous Ti_2_AlC, such as fracture toughness, flexural strength, and fatigue behavior.

However, the study of Ti_2_AlC foams with the highest porosity (93 vol.%) did not exhibit kinking or kink band formation but rather a typical brittle fracture characteristic of highly porous ceramic materials [35]. Nevertheless, a comprehensive understanding of the effect of porosity on deformation mechanisms as a function of porosity level requires further investigation. In particular, systematic studies should be conducted on materials containing large macropores combined with reduced microporosity within the load-bearing struts [35].

### 3.2. Elastic Modulus

**Figure 4:**
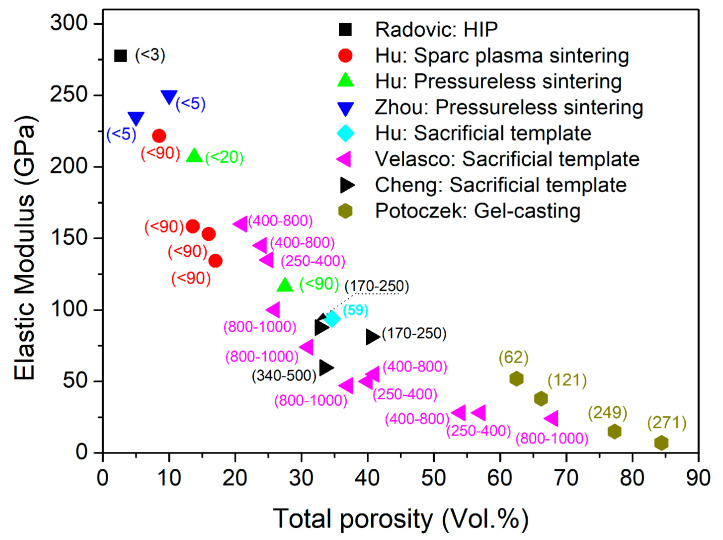
Reported elastic moduli of porous Ti_2_AlC as a function of total porosity. Numbers in parentheses indicate the average pore size (µm) or pore size range [27,28,31,37,40,41].

The elastic modulus of ceramics strongly depends on porosity, as an increase in porosity leads to a reduction in the overall stiffness of the material. Dense Ti_2_AlC exhibits a high elastic modulus of 277.6 GPa [5,8]. The elastic modulus values for porous Ti_2_AlC reported in the literature [27,28,31,37,40,41] are presented in Figure 4. The use of different fabrication techniques results in the formation of Ti_2_AlC with a broad porosity range of approximately 2–85 vol.%, which allows for tuning of the Young’s modulus (Figure 4). The elastic modulus decreases markedly with increasing total porosity, from 277 GPa at 2 vol.% to 7.0 GPa at 84.4 vol.%. In addition, the elastic modulus is influenced by pore size. For example, samples prepared by the sacrificial template method using saccharose with particle sizes in the range of 800–1000 µm exhibited a lower Young’s modulus (~100 GPa) compared to samples with smaller pore sizes ranging from 250 to 400 µm (~130 GPa) at a comparable porosity level (~25 vol.%) [31].

To predict the Young’s modulus of porous Ti_2_AlC based on the Young’s modulus of the fully dense material and the total porosity, several models commonly used for porous ceramics were applied, including the following:

Exponential model [42]:

(1) $$\frac{E}{E_{0}}=e^{-b\cdot P}$$

Hasselman model [43]:

(2) $$\frac{E}{E_{0}}=\frac{b\cdot P}{1+\left(b-1\right)\cdot P}$$

Composite spheres model [44,45]:

(3) $$\frac{E}{E_{0}}=\frac{{\left(1-P\right)}^{2}}{1+b\cdot P}$$

where *E*_0_ and *E* are the Young’s moduli of fully dense and porous Ti_2_AlC, *P* is the total porosity, and *b* is an empirical constant.

Hu et al. [28] and Velasco et al. [31] reported good agreement between the Young’s modulus values predicted by these three models and experimental data for porosity ranges of 3–35 vol.% and 3–70 vol.%, respectively. According to the data reported by Velasco et al. [31], the model that best fits the experimental data is the composite spheres model (*R*^2^ = 0.96), compared with the Hasselman model (*R*^2^ = 0.88) and the exponential model (*R*^2^ = 0.89). In contrast, according to the data reported by Hu et al. [28], the same correlation coefficient (*R*^2^ = 0.92) was obtained for all three of the above-mentioned models.

For higher porosity levels (68–82 vol.%), Fey et al. [36] analyzed the pore network using micro-computed tomography. Based on these data, the Young’s modulus was simulated using the finite element method (FEM), yielding results consistent with the experimental data [36].

### 3.3. Thermal Conductivity

**Figure 5:**
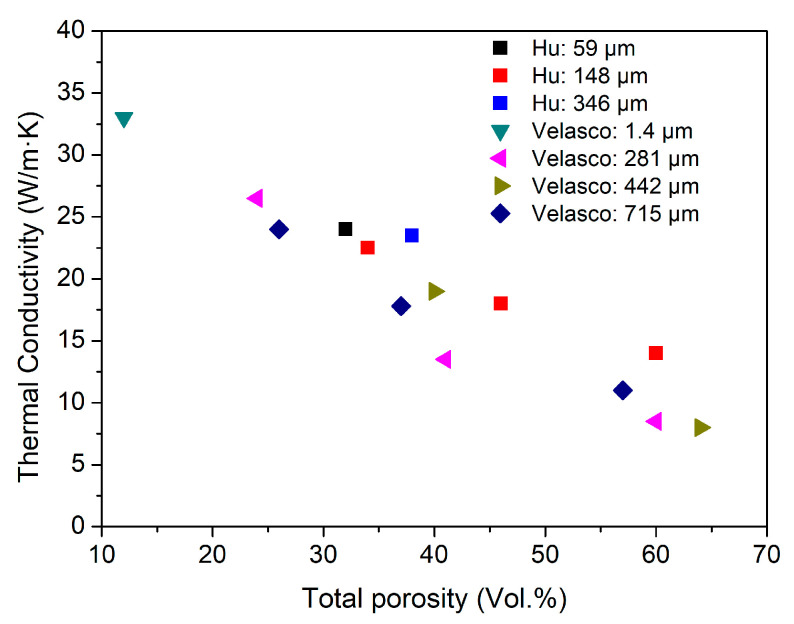
Reported room-temperature thermal conductivity of porous Ti_2_AlC as a function of total porosity. In the legend, the average pore size (µm) is indicated [28,31].

Dense Ti_2_AlC is considered a good thermal conductor, with a thermal conductivity of approximately 46 W/m·K [7,46,47]. In porous ceramics, heat transfer occurs predominantly through conduction within the solid framework separating the pores. Heat conduction through the pore interior is practically negligible due to the very low thermal conductivity of air (~0.02 W·m^−1^·K^−1^ at room temperature) [48]. Literature-reported thermal conductivities of porous Ti_2_AlC at room temperature as a function of total porosity are presented in Figure 5. Tsipas et al. and Hu et al. reported that the decrease in thermal conductivity with increasing porosity and pore size can be attributed to the increased volume fraction of air-filled pores, which act as thermal insulators [28,32]. In porous Ti_2_AlC, thermal conductivity is governed more by the total porosity than by the average pore size, as shown in the legend of Figure 5. However, the role of pore size cannot be neglected, as significant scatter in thermal conductivity values is observed, particularly within the porosity range of 30–50 vol.%, especially among results reported by different authors, although all authors employed the same technique for fabricating porous samples—the sacrificial template method [28,31].

**Figure 6:**
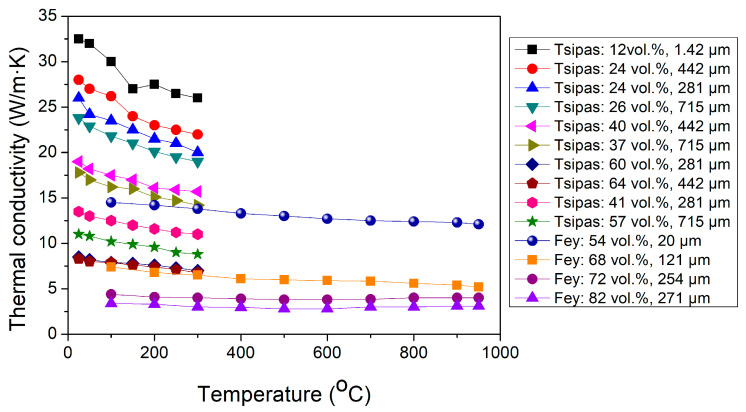
Reported thermal conductivity of porous Ti_2_AlC as a function of temperature. To the right of the graph, the porosity of the sample (vol%) and the average pore size (μm) are indicated [32,36].

The available literature data on the temperature dependence of the thermal conductivity of porous Ti_2_AlC are presented in Figure 6. The thermal conductivity decreases with increasing temperature. This behavior is attributed to enhanced phonon scattering resulting from increased lattice vibrations at elevated temperatures [32,36]. Although thermal conductivity is also influenced by pore size, its effect is less pronounced than that of temperature, as shown in the legend of Figure 6.

The strong tunability of thermal conductivity with porosity, pore size and temperature makes porous Ti_2_AlC attractive for heat exchangers and thermal management components.

### 3.4. High-Temperature Oxidation Resistance

Dense Ti_2_AlC exhibits excellent high-temperature oxidation resistance. This behavior is attributed to the selective oxidation of aluminum, which leads to the formation of a dense and protective α-Al_2_O_3_ scale [10,49]. It is well documented in the literature that heating elements made of dense Ti_2_AlC can withstand up to 8000 thermal shock cycles from temperatures as high as 1350 °C down to room temperature without sustaining any damage [16,18].

In porous materials, the oxidation rate is significantly higher than in their dense counterparts due to the presence of open and interconnected pores that facilitate oxidant diffusion into the substrate. Key factors governing the oxidation resistance of porous MAX phases include total and open porosity, pore size, pore connectivity, and strut thickness. Available literature data on the oxidation behavior of porous Ti_2_AlC are summarized in Table 2 [32,50]. Since Al, Cr, and Si are classified among the most oxidation-resistant metals in air because they exhibit selective oxidation and form dense, adherent, and slowly growing oxide scales [51], the literature data for porous Ti_3_SiC_2_ and Cr_2_AlC are also included in Table 2 [32,52,53,54].

Tsipas et al. studied the oxidation kinetics of Ti_2_AlC with a total porosity of 20 vol.% and Ti_3_SiC_2_ with a total porosity ranging from 20 to 60 vol.% [32]. The materials were resistant to oxidation and thermal shock in cyclic tests (10 cycles of 24h) at 1000 °C (Ti_2_AlC) and 900 °C (Ti_3_SiC_2_). The in situ formation of an outer TiO_2_ and an inner Al_2_O_3_ layer was responsible for the oxidation resistance of porous Ti_2_AlC. In the Ti_3_SiC_2_ case, an outer layer of TiO_2_ and an inner layer composed of a mixture of TiO_2_ and SiO_2_ were formed.

Potoczek et al. studied the high-temperature oxidation behavior of Ti_2_AlC gel-cast foams with a high total porosity of 87 vol.% in the temperature range of 600–1000 °C, in static air, and with exposure times of 6.5 h [50]. The foams were characterized by low weight gains during oxidation in the temperature range of 600–800 °C; however, at 1000 °C, they were completely oxidized after 1.5 h. Even after the complete oxidation of Ti_2_AlC to Al_2_O_3_ and TiO_2_ in air at 1000 °C, the macropore morphology remained open.

The oxidation behavior of Cr_2_AlC foams with total porosity ranging from 35 to 75 vol.% was investigated by Gonzalez-Julian et al. [52]. After 1 h oxidation tests at maximum temperatures from 800 to 1300 °C, Cr_2_AlC foams exhibited excellent oxidation resistance due to the formation of a thin and continuous protective outer layer composed of α-Al_2_O_3_. In subsequent studies on Cr_2_AlC foams with a total porosity of 53 vol.%, the exposure time at the maximum oxidation temperature was extended to 100 h [53]. Based on the results demonstrating the excellent oxidation resistance of these foams, a safety criterion was proposed. Specifically, it was suggested that Cr_2_AlC foams can be used at temperatures up to 1000 °C for a predicted lifetime of approximately 1000 h.

Wang and He [54] investigated the oxidation behavior of a reactively synthesized porous Ti_3_(Si,Al)C_2_ compound with a total porosity of 42.9 vol.% and a maximum pore size of 5.3 µm at 800 °C in ambient air. It was demonstrated that after 100 h of oxidation, the open porosity and permeability still retained high values [54].

The maximum temperatures applied for porous MAX phases were 1300 °C for Cr_2_AlC, 1000 °C for Ti_2_AlC, and 900 °C for Ti_3_SiC_2_ (Table 2). All investigated MAX phases exhibited good oxidation resistance at moderate temperatures. However, at 1000 °C, the best resistance was demonstrated by Cr_2_AlC foams with 53 vol.% porosity, with a predicted lifetime of approximately 1000 h. In addition to the oxidation temperature, the oxidation resistance strongly depended on total porosity. Ti_2_AlC foams with a total porosity of 20 vol.% exhibited good oxidation resistance at 1000 °C for up to 240 h (10 cycles of 24 h), whereas foams of the same material with a total porosity of 87 vol.% were completely oxidized after 1.5 h [32,50].

To expand knowledge of the high-temperature oxidation resistance of porous MAX phases, further studies with longer exposure times (>1 month) should be conducted. Moreover, these studies should also consider oxidizing atmospheres other than air.

### 3.5. Permeability

There is very limited information in the literature regarding the permeability of porous Ti_2_AlC [32,37]. However, in applications involving open-porosity ceramics, permeability is a crucial parameter, as it determines the transport properties of fluids or gases through interconnected pores within the material.

Tsipas et al. investigated the permeability of porous Ti_2_AlC fabricated using the sacrificial template method [32]. It was demonstrated that the Darcian permeability for samples with open porosity levels of 40 and 60 vol.% ranged from approximately 5 × 10^−17^ (for 40 vol.%) to ~8 × 10^−13^ m^2^ (for 60 vol.%). The non-Darcian permeability was not reported in that study [32]. While knowledge of Darcian permeability is sufficient for the design of porous ceramic components operating at low fluid or gas flow velocities, at higher flow rates it becomes necessary to consider both Darcian and non-Darcian permeability [23]. Similarly, Potoczek et al. studied the permeability of Ti_2_AlC foams produced via the gel-casting method, with open porosity ranging from 55.5 to 82.0 vol.% [37]. Within this porosity range, the Darcian permeability varied from 3.0 × 10^−12^ to 9.4 × 10^−10^ m^2^, while the non-Darcian permeability ranged from 3.9 × 10^−8^ to 3.4 × 10^−5^ m. The permeability values reported in previous studies are typical for highly porous ceramics and indicate that Ti_2_AlC with open porosity in the range of 40–80 vol.% can be used in a variety of demanding applications requiring fluid permeation [23,32,37]. These include pressure-assisted metal infiltration for the fabrication of metal/ceramic composites, catalytic treatment of engine exhaust gases, and heat exchangers.

### 3.6. Electrical Resistivity

Owing to the coexistence of metallic and covalent bonds, Ti_2_AlC is considered a very good electrical conductor, exhibiting an electrical resistivity in the range of 0.23–0.40 µΩ·m at room temperature [4,8,16]. To date, the electrical resistivity of porous Ti_2_AlC as a function of porosity and temperature has only been investigated by Tsipas et al., using samples fabricated via the sacrificial template method [32]. Available data indicate that the electrical resistivity of porous Ti_2_AlC increases with increasing temperature, total porosity, and pore size [32].

In the temperature range of 25–550 °C, the electrical resistivity of porous Ti_2_AlC with a total porosity of 12 vol.% and an average pore size of 1.42 µm increased from 0.3 to 1.0 µΩ·m. With increasing porosity and pore size (57 vol.%, 715 µm), the electrical resistivity increased from 1.8 µΩ·m (25 °C) to 4.1 µΩ·m (550 °C) [32]. It has been reported that increasing porosity leads to a higher volume fraction of pores, which act as electrical insulators; consequently, the electrical resistivity increases with increasing porosity. Furthermore, an increase in pore size reduces the effective cross-sectional area available for electron transport, resulting in an additional increase in electrical resistivity [32].

It should be noted that the electrical resistivity data for porous Ti_2_AlC reported in the literature often correspond to multiphase materials rather than phase-pure Ti_2_AlC [32]. In many cases, the samples consist predominantly of Ti_2_AlC accompanied by secondary phases such as Ti_3_AlC_2_, TiC, and Al_2_O_3_. This is mainly due to the limited availability of commercially pure MAX-phase powders, as well as partial thermal decomposition of porous Ti_2_AlC to Ti_3_AlC_2_ and TiC during pressureless sintering [28,35]. To fully elucidate the effect of porosity on electrical resistivity, further studies using higher-purity porous Ti_2_AlC are required. Additionally, the investigated range of porosity and pore architectures should be expanded.

## 4. Potential Applications of Porous Ti_2_AlC and Related MAX Phases

Although dense MAX phases have been intensively investigated for more than 30 years and over 150 MAX phases have been identified to date, their industrial implementation remains limited. To the best of the authors’ knowledge, the only current large-scale industrial application of MAX phases is in pantograph components incorporating Cu/Ti_3_AlC_2_ cermets for high-speed railways in China [55,56,57]. The limited commercialization of dense MAX phases is primarily associated with the restricted availability of high-purity powders at acceptable costs [18]. At present, therefore, only potential applications of porous Ti_2_AlC can be discussed. The potential applications of porous Ti_2_AlC reported in the literature are summarized in Table 3. Related MAX phases are also included in Table 3 to illustrate the broad application spectrum of porous MAX phases.

### 4.1. Automobile Industry–Environmental Protection

Potential applications of porous MAX phases (Ti_2_AlC and Ti_3_AlC_2_) in the automotive industry include honeycomb structures and foam-based catalyst supports. Fang et al. demonstrated an electrically conductive Ti_2_AlC honeycomb with channel sizes of 1000 µm and micropore sizes of 2–15 µm monoliths that can be used as a diesel particulate filter [38]. Due to its electrical conductivity, the honeycomb structure can be resistively heated during engine cold start, thereby mitigating the cold-start problem.

Ti_3_AlC_2_ foam, with a total porosity of 80 vol.%, fabricated by the polymer replication technique, was used as a support for a CeO_2_ nanostructured catalyst in automotive exhaust systems [58]. Similarly, the electrical conductivity of the MAX-phase foam enables resistive heating at low engine temperatures, improving catalyst performance during cold start.

### 4.2. Filters for Zn Metallurgy

Ti_3_SiC_2_ filters manufactured by reactive sintering were investigated by Liu et al. for Zn metallurgy [59]. The open porosity of this material was 48–55%, and the pore size ranged from 3 to 10 µm. Owing to its excellent corrosion resistance in concentrated acids, porous Ti_3_SiC_2_ exhibited high filtration efficiency in ZnSO_4_ solutions. Laboratory-scale tests demonstrated a reduction in zinc powder consumption by approximately 40% and in electricity consumption by 8%, while yielding the highest-grade electrolytic zinc.

### 4.3. Advanced Thermal Management Systems

Ti_3_(Si,Al)C_2_, produced by reactive sintering, has been proposed as a wick material for loop heat pipes by Cao et al. [60]. The material was characterized by a total porosity of 30 vol.%, micropore size of 2.46 µm, and macropore size of 7.24 µm. Materials used in loop heat pipes must exhibit high capillary pumping capability, good thermal shock resistance, chemical and oxidation resistance, and good machinability. The intrinsic combination of thermal conductivity, oxidation resistance, and tailored porosity makes porous MAX phases promising candidates for advanced thermal management systems.

### 4.4. Light Creep Resistance Refractory Materials

Araki et al. demonstrated very good creep resistance of Cr_2_AlC foams with a total porosity of 53 and 75 vol.% in the temperature range of 800–1200 °C [61]. The creep rates of porous Cr_2_AlC with a total porosity of 53 vol.% under 5 MPa at 1000 °C were 2.2 × 10^−7^ s^−1^ during heating and 5.0 × 10^−9^ s^−1^ during cooling. Consequently, porous Cr_2_AlC has been proposed as a lightweight refractory material capable of sustaining loads for long periods without significant deformation and exhibiting a low creep rate at high temperatures.

### 4.5. Membrane Supports for H_2_ Cleaning

Ti_3_AlC_2_ doped with Al_2_O_3_ has been investigated as a membrane support for hydrogen purification [62]. Membrane supports require high mechanical strength, sufficient permeability, and high resistance to hydrogen embrittlement. Kusharov et al. fabricated Ti_3_AlC_2_/Al_2_O_3_ composites with a Ti_3_AlC_2_/Al_2_O_3_ ratio in the range of 1/10–1/2 [62]. Depending on the Ti_3_AlC_2_ content, composites with open porosity ranging from 3.4 to 40 vol.% and pore sizes between 0.5 and 4 µm were obtained. The maximum hydrogen flux for composites with an open porosity of 40 vol.% ranged from 30 to 200 mol·m^−2^·s^−1^ depending on hydrogen pressure and working temperature.

### 4.6. Electrodes for H_2_ Evolution Reaction

High-entropy MAX phases constitute a new branch of MAX-phase materials. A characteristic feature of these materials is the random distribution of several metals (≥4) at the M-site together with high configurational entropy, which stabilizes the structure. Compared with conventional MAX phases, high-entropy MAX phases exhibit improved resistance to aqueous corrosion and high-temperature oxidation, as well as enhanced thermal stability [71,72]. High-entropy porous (20–23 vol.%) V_2_Sn_x_(FeCoNi)_1.2−x_C MAX phases produced by reactive sintering were investigated as electrode materials for the hydrogen evolution reaction (HER) [63]. HER is a key reaction enabling green hydrogen production from renewable energy sources and represents a cornerstone of the future hydrogen economy. These porous MAX phases exhibited excellent catalytic activity with an overpotential of 284 mV at 20 mA cm^−2^ and favorable Tafel slopes of −0.26 V in alkaline media. Their performance was attributed to the combination of open porosity and chemical stability. V_2_Sn_x_(FeCoNi)_1.2−x_C also demonstrated excellent chemical stability in 6 M KOH solution for 13 h [63]. In addition, high-entropy porous MAX phases may find further applications in heterogeneous catalysis.

### 4.7. Hydrogen Storage Materials

De and Bhattacharyya synthesized TiVAl_x_C MAX phases with varying Al molar content and sintering temperatures to optimize hydrogen storage properties [64]. The materials were synthesized using molten-salt-shielded synthesis, a method that employs molten salts as both the reaction medium and a protective layer. Depending on the Al molar fraction (1.1–1.5), the pore size ranged from 2.5 to 9.5 nm, while the pore volume was between 0.00821 and 0.02624 cm^3^ g^−1^. The gravimetric hydrogen storage capacities of TiVAl_1_._3_C and V_2_AlC phases were 0.62 and 1.29 wt.%, respectively, under an Ar/H_2_ (95/5) gas flow at 450 °C with a holding time of 2 h and a gas flow rate of 100 mL min^−1^ [64].

### 4.8. Lightweight Interpenetrating Metal/MAX Phase Composites

Porous Ti_2_AlC and Ti_3_SiC_2_ have been used as preforms for molten metal infiltration to produce interpenetrating phase composites (IPCs). These materials are characterized by a unique three-dimensional architecture consisting of interpenetrating ceramic and metal skeletons throughout the entire structure. Newnham classified such materials as (3–3) composites [65]. In practice, the most common approach for producing these composites is infiltration of liquid metal into a porous ceramic body, called a preform, characterized by interconnected porosity. Since this review focuses on lightweight materials, the discussion of metallic components is limited to light metals such as Al, Mg, and their alloys.

Hu et al. fabricated porous Ti_2_AlC foams using the sacrificial template method with a total porosity of 39.9–41.6% and pore sizes of 42–83 µm, 77–276 µm, and 167–546 µm [66]. The porous foams were subsequently pressure-infiltrated with a 6061 Al alloy. The reported compressive strength for the composite with a Ti_2_AlC/Al alloy volume ratio of 27/73 was approximately 1100 MPa [66].

Amini et al. investigated composites containing 50 vol.% Ti_2_AlC and nanocrystalline Mg (50–100 nm) [67]. Porous Ti_2_AlC preforms (~50 vol.%) were produced by pressing followed by incomplete sintering. The composites were fabricated by pressureless infiltration. The tensile strength reached 350 MPa, while the compressive strength was approximately 700 MPa. In addition, a significant improvement in damping properties was observed [68].

Porous Ti_2_AlC preforms prepared using incomplete sintering were also infiltrated with magnesium alloys AZ31, AZ61, and AZ91 [69]. Depending on the initial Ti_2_AlC powder particle size (0.5 or 0.9 µm) and the type of magnesium alloy used, the compressive strength ranged from 668 to 773 MPa.

Zhou et al. produced Ti_3_SiC_2_ with total porosity ranging from 10 to 30 vol.% using the sacrificial template method [70]. The porous samples were subsequently infiltrated with a 6061 Al alloy. High compressive strengths (743–932 MPa) were reported for Ti_3_SiC_2_/6061Al composites containing 10–30 vol.% of the Al alloy.

Due to the superior mechanical properties of IPCs containing MAX phases, many advanced technological applications have been proposed. Examples include pantographs for high-speed railways, missile tail structures, satellite antennas for space applications, disc impellers and brake discs in the automotive industry, as well as sporting equipment such as bicycles and tennis rackets [73].

## 5. Conclusions and Future Outlook

This review summarizes the current state of knowledge on the processing, structure–property relationships, and potential applications of porous Ti_2_AlC.

The available, albeit relatively limited, literature demonstrates that various fabrication methods—such as incomplete sintering, sacrificial templating, replication of polyurethane sponges, gel casting of foams, extrusion of honeycombs, and direct ink writing—enable the production of porous Ti_2_AlC with average pore sizes ranging from a few micrometers to several millimeters and total porosity ranging from a few to 96 vol.%.

Total porosity has a significant influence on the compressive strength, elastic modulus, thermal conductivity, and high-temperature oxidation resistance of porous Ti_2_AlC. Depending on the total porosity level, the compressive strength ranges from 430 MPa (16 vol.%) to 1.6 MPa (93 vol.%). The elastic modulus decreases with increasing total porosity, from 277 GPa at 2 vol.% to 7.0 GPa at 84.4 vol.%, while the thermal conductivity decreases from 33 W/m·K (2 vol.%) to 8 W/m·K (64 vol.%) with increasing porosity. Within the moderate temperature range of 700–800 °C, highly porous Ti_2_AlC exhibits good resistance to high-temperature oxidation. However, at 1000 °C, Ti_2_AlC foams with a total porosity of 20 vol.% maintain good oxidation resistance for up to 240 h (10 cycles of 24 h), whereas foams of the same material with a total porosity of 87 vol.% are completely oxidized after 1.5 h.

The available literature indicates that pore size has a comparatively smaller effect on the mechanical and physicochemical properties than total porosity. However, the role of pore size and morphology requires further clarification.

Potential applications of porous Ti_2_AlC and related MAX phases include electrically conductive diesel particulate filter (DPF) systems and catalyst supports in the automotive industry, filters for zinc metallurgy, advanced thermal management systems, lightweight creep-resistant refractory materials, and lightweight interpenetrating metal/MAX phase composites.

Moreover, porous MAX phases are promising materials for the hydrogen economy, including applications such as membrane supports for H_2_ purification, electrodes for hydrogen evolution, and hydrogen storage materials.

Future research should focus on several key areas:

Achieving high phase purity of Ti_2_AlC powders at an acceptable cost, as well as further investigation of the mechanical and physicochemical properties of porous structures obtained using various techniques, is particularly important given that most of the currently available literature concerns the sacrificial template method.

Conducting additional mechanical studies, not only at room temperature but also at elevated temperatures;

Expanding the understanding of the high-temperature oxidation resistance of porous MAX phases through long-term studies (exceeding one month), including investigations in oxidizing atmospheres other than air, such as H_2_–H_2_S and water vapor; additionally, evaluating the thermal shock behavior of porous Ti_2_AlC during cooling in air and water.

Further research on the fabrication and properties of lightweight MAX phase/metal composites produced by metal infiltration into porous preforms fabricated using various techniques is necessary, given their wide range of potential applications.

Exploring high-entropy porous MAX phases, particularly with respect to their potential applications in the hydrogen economy, as well as in the broadly defined field of catalysis.

Despite the broad range of potential research directions, several challenges appear particularly critical for the further development and practical implementation of porous Ti_2_AlC and related MAX phases:

Among the identified research directions, the development of architected porous Ti_2_AlC structures fabricated by direct ink writing (DIW) appears particularly important. Compared with conventional stochastic foams produced by sacrificial templating or direct foaming methods, DIW enables precise control of pore architecture, strut geometry, and hierarchical porosity across multiple length scales, thereby offering new opportunities for tailoring the mechanical, thermal, and transport properties of porous MAX phases. Such structures may be especially attractive for advanced applications in thermal management systems, catalyst supports, lightweight high-temperature components, and multifunctional energy-related devices.

In parallel, further progress in the field will require the development of advanced numerical approaches capable of describing both stochastic foam-like structures and periodic architected porous materials produced by additive manufacturing techniques. In particular, multiscale modeling of hierarchical porous structures may significantly improve the understanding of structure–property relationships and accelerate the optimization of porous MAX phases for new applications.

## Figures and Tables

**Table 1:** Manufacturing of porous Ti_2_AlC by different methods.

Method	Main Processing Parameters	Total Porosity Open Porosity Pore Size	Ref.
Incomplete sintering	Forming the pellets by pressing Reducing the sintering temperature and/or the sintering time	Up to several vol.% n.r. A few µm	[27]
Sacrificial template	Mixing Ti2AlC with space holder powders Cold pressing Dissolution of the space holder, drying, and sintering at 1400 °C	20–70 vol.% 15–65 vol.% 40–1000 µm	[28,29,30,31,32,33]
Replica method	Preparation of Ti2AlC with modificatory additives Impregnation of the polyurethane foam Removing the excess slip and drying Foam pyrolysis at 800 °C, sintering at 1400 °C	Vol.%-n.r., Open porosity is predominant 10 ppi	[34]
Gel casting of foams	Preparation of Ti2AlC slurry Mixing Ti2AlC slurry with agarose solution at 60 °C Adding surfactants and mechanical foaming Gelation, drying, and sintering at 1400 °C	50–93 vol.% 40–92 vol.% 20–615 µm	[35,36,37]
Honeycomb extrusion	Preparation of plastic feed material for extrusion containing Ti, Al, graphite powders, water, and organic additives Extrusion, drying Isothermal treatment at 650 °C to dissipate the latent heat from reactions between Ti, Al and C powders; sintering at 1400 °C	Channel size: 1000 µm Open porosity is predominant Micropore size: 2–15 µm	[38]
Direct ink writing (DIW)	Preparation of Ti2AlC paste with the binder (polyethylene glycol, polyvinyl alcohol). Extrusion through a conical nozzle Obtaining filaments for building tetragonal lattices Drying, sintering at 1400 °C	44, 57 and 63 vol.% Open porosity is predominant Pore size as a spacing between filaments: 1200, 1600 and 2400 µm	[39]

Abbreviations: n.r.: not reported; Ref.: references.

**Table 2:** Summary of high-temperature oxidation of porous MAX phases containing Al, Si or Cr.

Porous MAX Phase	Manufacturing Method	Total Porosity and Pore Size	Oxidation Conditions	Scale Composition	Ref.
Ti_2_AlC	Sacrificial template: saccharose as the space holder, isostatic pressing at 400 MPa, pressureless sintering	20 vol.% 250–400 µm	Cyclic T = 1000 °C t = 10 cycles of 24 h	TiO_2_ (rutile), α-Al_2_O_3_	[32]
Ti_2_AlC	Gel casting of foams, pressureless sintering	87 vol.% 335 ± 138 µm	Continuous T = 600–1000 °C t = 6.5 h	TiO_2_ (anatase), TiO_2_ (rutile) and α-Al_2_O_3_	[50]
Ti_3_SiC_2_	Sacrificial template: saccharose as the space holder, isostatic pressing at 400 Mpa, pressureless sintering	20–60 vol.% 250–1000 µm	Cyclic T =900 °C t = 10 cycles of 24 h,	SiO_2_ (β-tridymite) TiO_2_ (rutile)	[32]
Cr_2_AlC	Sacrificial template, NH_4_HCO_3_ as the space holder, uniaxial pressing at 200 MPa pressureless sintering	35–75 vol.% 90–400 µm	Continuous T = 800–1300 °C t = 1 h, heating rate to oxidation temperature: 10 °C/min	α-Al_2_O_3_ as the major phase and small amounts of Cr_7_C_3_ and Cr_3_C_2_	[52]
Cr_2_AlC	Sacrificial template, NH_4_HCO_3_ as the space holder, uniaxial pressing at 200 MPa, pressureless sintering	53 vol.% 180–250 µm	Continuous T = 900–1200 °C t = up to 100 h	Al_2_O_3_ as the major phase and small amounts of Cr_7_C_3_ and Cr_3_C_2_	[53]
Ti_3_(Si,Al)C_2_	Reaction synthesis of elemental powders	42.9 vol.% 5.3 µm	Continuous T = 800 °C t = 100 h	TiO_2_ (rutile) Al_3_Ti_5_O_2_	[54]

**Table 3:** Potential applications of porous MAX phases.

MAX Phase	Processing Route	Application	Required Properties	Ref.
Ti_2_AlC	Extrusion, drying, reactive sintering	Conductive honeycomb in automobile	Good thermal stability, high mechanical strength, good erosion resistance, low heat capacity, good thermal shock resistance, electrical conductivity	[38]
Ti_3_AlC_2_/CeO_2_	Replication of a polymeric foam, sintering, CeO_2_ deposition	Catalyst for gas exhaust devices in automobile	Good thermal stability, high mechanical strength, good erosion resistance, low heat capacity, good thermal shock resistance, electrical conductivity	[58]
Ti_3_SiC_2_	Reactive sintering	Filters for Zn(SO_4_)_2_ solutions	Permeability, corrosion resistance in concentrated acids	[59]
Ti_3_(Al,Si)C_2_	Sacrificial template, pressureless sintering	Loop heat pipes	Large capillary pumping capability, good thermal shock resistance, chemical and oxidation resistance, good machinability	[60]
Cr_2_AlC	Sacrificial template, pressureless sintering	Light refractory material with high creep resistance	Ability to carry loads for long periods of time without significant deformation,	[61]
Ti_3_AlC_2_/Al_2_O_3_	Slip casting, drying, pressureless sintering	Membrane support for hydrogen cleaning	Sufficient mechanical strength, permeability	[62]
V_2_Sn_x_(FeCoNi)_1.2−x_C (x = 0.4–0.8)	Reactive sintering	Electrodes for H_2_ evolution	Chemical resistance in alkaline solutions	[63]
TiVAl_x_C (x = 1.1–1.5)	Molten-salt-shielded synthesis	Hydrogen storage	Elevated operating temperature, several wt.% hydrogen capacity, reversible adsorption/desorption	[64]
Ti_2_AlC Ti_3_SiC_2_	Sacrificial template, pressureless sintering	Preforms for interpenetrating phase composites	Accepted compression strength, open porosity	[64,65,66,67,68,69,70]

## Data Availability

No new data were created or analyzed in this study. Data sharing is not applicable to this article.

## References

[1] Nowotny V.H. (1971). Strukturchemie Einiger Verbindungen der Übergangsmetalle mit den Elementen C, Si, Ge, Sn. Prog. Solid State Chem..

[2] Nowotny V.H., Schuster J.C., Rogl P. (1982). Structural Chemistry of Complex Carbides and Related Compounds. J. Solid State Chem..

[3] Barsoum M.W., El-Raghy T. (1996). Synthesis and Characterization of a Remarkable Ceramic Ti_3_SiC_2_. J. Am. Ceram. Soc..

[4] Barsoum M.W. (2013). MAX Phases: Properties of Machinable Ternary Carbides and Nitrides.

[5] Barsoum M.W., Radovic M. (2011). Elastic and Mechanical Properties of the MAX Phases. Annu. Rev. Mater. Res..

[6] Barsoum M.W., Ali M., El-Raghy T. (2000). Processing and characterization of Ti_2_AlC, Ti_2_AlN, and Ti_2_AlC_0_._5_N_0_._5_. Metall. Mater. Trans. A.

[7] Hettinger J.D., Lofland S.E., Finkel P., Meehan T., Palma J., Harrell K., Gupta S., Ganguly A., El-Raghy T., Barsoum M.W. (2005). Electrical Transport, Thermal Transport, and Elastic Properties of M_2_AlC (M = Ti, Cr, Nb, and V). Phys. Rev. B.

[8] Wang X.H., Zhou Y.C. (2010). Layered Machinable and Electrically Conductive Ti_2_AlC and Ti_3_AlC_2_ Ceramics: A Review. J. Mater. Sci. Technol..

[9] Li Z., Zhang Y., Wang K., Wang Z., Ma G., Ke P., Wang A. (2024). Highly Dense Passivation-Enhanced Corrosion Resistance of Ti_2_AlC MAX Phase Coating in 3.5 wt.% NaCl Solution. Corros. Sci..

[10] Wang X.H., Zhou Y.C. (2003). High-Temperature Oxidation Behavior of Ti_2_AlC in Air. Oxid. Met..

[11] Xiao J., Yang T., Wang C., Xue J., Wang Y. (2015). Investigations on Radiation Tolerance of M_n+1_AX_n_ Phases: Study of Ti_3_SiC_2_, Ti_3_AlC_2_, Cr_2_AlC, Cr_2_GeC, Ti_2_AlC, and Ti_2_AlN. J. Am. Ceram. Soc..

[12] Su R., Zhang H., Liu L., Shi L., Wen H. (2021). Reversible phase transformation in Ti_2_AlC films during He radiation and subsequent annealing. J. Eur. Ceram. Soc..

[13] Suh M., Lee D.H., Sloof W.G., Lee K.S. (2024). Effect of Temperature on the Healing Capacity and Mechanical Properties of Ti_2_AlC MAX Phase Ceramics. Int. J. Appl. Ceram. Technol..

[14] Alam M.S., Chowdhury M.A., Khandaker T., Hossain M.S., Islam M.S., Islam M.M., Hasan M.K. (2024). Advancements in MAX Phase Materials: Structure, Properties, and Novel Applications. RSC Adv..

[15] Sun Z.M. (2011). Progress in Research and Development on MAX Phases: A Family of Layered Ternary Compounds. Int. Mater. Rev..

[16] Radovic M., Barsoum M.W. (2013). MAX Phases: Bridging the Gap Between Metals and Ceramics. Am. Ceram. Soc. Bull..

[17] Chen X., Be G. (2017). Toughening Mechanisms in Nanolayered MAX Phase Ceramics—A Review. Materials.

[18] Gonzalez-Julian J. (2021). Processing of MAX Phases: From Synthesis to Applications. J. Am. Ceram. Soc..

[19] Colombo P. (2008). In Praise of Pores. Science.

[20] Gibson L.J., Ashby M.F. (1997). Cellular Solids: Structure and Properties.

[21] Hammel E.C., Ighodaro O.R., Okoli O.I. (2014). Processing and Properties of Advanced Porous Ceramics: An Application-Based Review. Ceram. Int..

[22] Ohji T., Fukushima M. (2012). Macro-Porous Ceramics: Processing and Properties. Int. Mater. Rev..

[23] Scheffler M., Colombo P. (2005). Cellular Ceramics: Structure, Manufacture, Properties and Applications.

[24] Colombo P. (2006). Conventional and Novel Processing Methods for Cellular Ceramics. Philos. Trans. R. Soc. A..

[25] Studart A.R., Gonzenbach U.T., Tervoort E., Gauckler L.J. (2006). Processing Routes to Macroporous Ceramics: A Review. J. Am. Ceram. Soc..

[26] Liu Q., Zhai W. (2022). Hierarchical Porous Ceramics with Distinctive Microstructures by Emulsion-Based Direct Ink Writing. ACS Appl. Mater. Interfaces.

[27] Zhou A.G., Barsoum M.W., Basu S., Kalidindi S.R., El-Raghy T. (2006). Incipient and Regular Kink Bands in Fully Dense and 10 vol.% Porous Ti_2_AlC. Acta Mater..

[28] Hu L., Benitez R., Basu S., Karaman I., Radovic M. (2012). Processing and Characterization of Porous Ti_2_AlC with Controlled Porosity and Pore Size. Acta Mater..

[29] Velasco B., Gordo E., Tsipas S.A. (2015). MAX Phase Ti_2_AlC Foams Using a Leachable Space-Holder Material. J. Alloys Compd..

[30] Velasco B., Tsipas S.A., Ferrari B., Gordo E. (2015). MAX Phase Foams Produced via Powder Metallurgy Process Using Water Soluble Space Holder. Powder Metall..

[31] Velasco B., Gordo E., Hu L., Radovic M., Tsipas S.A. (2018). Influence of Porosity on Elastic Properties of Ti_2_AlC and Ti_3_SiC_2_ MAX Phase Foams. J. Alloys Compd..

[32] Tsipas S.A., Tabares E., Weissgaerber T., Hutsch T., Sket F., Velasco B. (2021). Thermophysical Properties of Porous Ti_2_AlC and Ti_3_SiC_2_ Produced by Powder Metallurgy. J. Alloys Compd..

[33] Gonzalez-Julian J., Bram M. (2016). Processing and Characterization of Porous Ti_2_AlC Using Space Holder Technique. Key Eng. Mater..

[34] Bowen C.R., Thomas T. (2015). Macro-Porous Ti_2_AlC MAX-Phase Ceramics by the Foam Replication Method. Ceram. Int..

[35] Potoczek M., Guzi de Moraes E., Colombo P. (2015). Ti_2_AlC Foams Produced by Gel-Casting. J. Eur. Ceram. Soc..

[36] Fey T., Stumpf M., Chmielarz A., Colombo P., Greil P., Potoczek M. (2018). Microstructure, Thermal Conductivity and Simulation of Elastic Modulus of MAX-Phase (Ti_2_AlC) Gel-Cast Foams. J. Eur. Ceram. Soc..

[37] Potoczek M., Chmielarz A., Innocentini M.D.M., Silva I.C.P., Colombo P., Winiarska B. (2018). Porosity Effect on Microstructure, Mechanical and Fluid Dynamic Properties of Ti_2_AlC by Direct Foaming and Gel-Casting. J. Am. Ceram. Soc..

[38] Fang X., Wang X., Zhang H., Li Z., Li J., Zhou Y. (2015). A Cost-Efficient Fabrication Strategy for Conductive Ti_2_AlC Honeycomb Monolith Using Elemental Powders. Adv. Eng. Mater..

[39] Elsayed H., Chmielarz A., Potoczek M., Fey T., Colombo P. (2019). Direct Ink Writing of Three-Dimensional Ti_2_AlC Porous Structures. Addit. Manuf..

[40] Cheng F., Kim S.-M., Reddy J.N. (2015). Computational Modelling of the Plastic-Damage Behavior of Porous MAX Phase With Aligned Ellipsoid-Like Pores Under Uniaxial Compression. Int. J. Solids Struct..

[41] Radovic M., Barsoum M.W., Ganguly A., Zhen T., Finkel P., Kalidindi S.R., Lara-Curzio E. (2006). On the Elastic Properties and Mechanical Damping of Ti_3_SiC_2_, Ti_3_GeC_2_, Ti_3_Si_0.5_Al_0.5_C_2_ and Ti_2_AlC in the 300–1573 K Temperature Range. Acta Mater..

[42] Spriggs R.M. (1961). Expression for Effect of Porosity on Elastic Modulus of Polycrystalline Refractory Materials, Particularly Aluminium Oxide. J. Am. Ceram. Soc..

[43] Hasselman D.P.H. (1962). On the Porosity Dependence of the Elastic Moduli of Polycrystalline Refractory Materials. J. Am. Ceram. Soc..

[44] Ramakrishnan N., Arunachalam V.S. (1990). Effective Elastic Moduli of Porous Solids. J. Mater. Sci..

[45] Ramakrishnan N., Arunachalam V.S. (1993). Effective Elastic Moduli of Porous Ceramic Materials. J. Am. Ceram. Soc..

[46] Barsoum M.W., Salama I., El-Raghy T., Golczewski J., Porter W.D., Wang H., Seifert H.J., Aldinger F. (2002). Thermal and Electrical Properties of Nb_2_AlC, (Ti,Nb)_2_AlC and Ti_2_AlC. Metall. Mater. Trans. A.

[47] Bai Y., He X., Zhu C., Chen G. (2012). Microstructures, Electrical, Thermal, and Mechanical Properties of Bulk Ti_2_AlC Synthesized by Self-Propagating High-Temperature Combustion Synthesis with Pseudo Hot Isostatic Pressing. J. Am. Ceram. Soc..

[48] Kannuluik W.G., Carman E.H. (1951). The Temperature Dependence of the Thermal Conductivity of Air. Aust. J. Sci. Res. Ser. A Phys. Sci..

[49] Yu W., Vallet M., Levraut B., Gauthier-Brunet V., Dubois S. (2020). Oxidation Mechanisms in Bulk Ti_2_AlC: Influence of the Grain Size. J. Eur. Ceram. Soc..

[50] Potoczek M., Dąbek J., Brylewski T. (2023). Oxidation Behavior of Ti_2_AlC MAX-Phase Foams in the Temperature Range of 600–1000 °C. J. Therm. Anal. Calorim..

[51] Kofstad P. (1966). High Temperature Oxidation of Metals.

[52] Gonzalez-Julian J., Onrubia S., Bram M., Broeckmann C., Vassen R., Guillon O. (2018). High-Temperature Oxidation and Compressive Strength of Cr_2_AlC MAX Phase Foams with Controlled Porosity. J. Am. Ceram. Soc..

[53] Araki W., Matsumoto A., Arai Y., Yamada N., Malzbender J., Gonzalez-Julian J. (2020). Lifetime Estimation of Cr_2_AlC MAX Phase Foam Based on Long-term Oxidation and Fracture Mechanisms. Materialia.

[54] Wang Z., Jiang Y., He Y. (2019). Oxidation Behavior of Reactively Synthesized Porous Ti_3_(Si,Al)C_2_ Compound at 800 °C in Ambient Air. Ceram. Int..

[55] Zhai H.X., Ai M.X., Huang Z.Y., Zhou Y., Li S.B., Zhang Z.L. (2007). Unusual Microstructures and Strength Characteristics of Cu/Ti_3_AlC_2_ Cermet. Key Eng. Mater..

[56] Ai M.X., Zhai H.X., Huang Z.Y. (2007). Interformational Exfoliation of Ti_3_AlC_2_ Induced by Cu. Key Eng. Mater..

[57] Huang Z.Y., Zhai H.X., Ai M.X. (2007). A New Cu-Ti_3_AlC_2_ Cermet Exhibiting Excellent Tribological Properties. Key Eng. Mater..

[58] Sun Z., Liang Y., Li M., Zhou Y. (2010). Preparation of Reticulated MAX-Phase Support with Morphology-Controllable Nanostructured Ceria Coating for Gas Exhaust Catalyst Devices. J. Am. Ceram. Soc..

[59] Liu X., Zhang H., Jiang Y., He Y. (2015). Characterization and Application of Porous Ti_3_SiC_2_ Ceramic Prepared Through Reactive Synthesis. Mater. Des..

[60] Cao Y., Guo C., Yu Y., Ma J., Wu D., Zou Y. (2022). Performances of Loop Heat Pipe with the Novel Bi-Porous Quaternary MAX Phase Ti_3_(Al,Si)C_2_ Capillary Wick. Vacuum.

[61] Araki W., Gonzalez-Julian J., Malzbender J. (2019). High Temperature Compressive Creep of Dense and Porous Cr_2_AlC in Air. J. Eur. Ceram. Soc..

[62] Kashkarov E., Krinitcyn M., Dyussambayev A., Pirozhkov A., Koptsev M. (2023). Structure and Properties of Porous Ti_3_AlC_2_-Doped Al_2_O_3_ Composites Obtained by Slip Casting Method for Membrane Application. Materials.

[63] Yang J., Fan Y., Liu Y., Zhang C., Zou H., Xiong L., Li X. (2022). Self-supporting Porous High-Entropy MAX Electrode for Highly Active Electrocatalyst H_2_ Evolution in Alkali Solution. J. Porous Mater..

[64] De S.K., Bhattacharyya S. (2023). Hydrogen Adsorption by a Porous Bimetallic Solid Solution Carbide MAX Phases Synthesized in an Open Atmosphere. J. Energy Storage.

[65] Newnham R.F., Skinner D.P., Cross L.E. (1978). Connectivity and Piezoelectric-Pyroelectric Composites. Mater. Res. Bull..

[66] Hu L., O’Neil M., Erturun V., Benitez R., Proust G., Karaman I., Radovic M. (2016). High-Performance Metal/Carbide Composites with Far-From-Equilibrium Compositions and Controlled Microstructures. Sci. Rep..

[67] Amini S., Ni C., Barsoum M.W. (2009). Processing, Microstructural Characterization and Mechanical Properties of a Ti_2_AlC/Nanocrystalline Mg-Matrix Composite. Compos. Sci. Technol..

[68] Kontsos A., Loutas T., Kostopoulos V., Hazeli K., Anasori B., Barsoum M.W. (2011). Nanocrystalline Mg–MAX Composites: Mechanical Behavior Characterization via Acoustic Emission Monitoring. Acta Mater..

[69] Anasori B., Caspi E.N., Barsoum M.W. (2014). Fabrication and Mechanical Properties of Pressureless Melt Infiltrated Magnesium Alloy Composites Reinforced with TiC and Ti_2_AlC Particles. Mater. Sci. Eng. A.

[70] Zhou C., Wu X., Ngai T.L., Li L., Ngai S., Chen Z. (2018). Al Alloy/Ti_3_SiC_2_ Composites Fabricated by Pressureless Infiltration with Melt-Spun Al Alloy Ribbons. Ceram. Int..

[71] Tian Z., Hu F., Zhang P., Fan Y., Shamshirgar A.S., Wu S., Cai L., Bai Y., Wu X., Rosen J. (2025). High-Entropy Engineering of A-Site in MAX Phases Toward Superior Microwave Absorption Properties. Matter.

[72] Guo M., Cao G., Pan H., Guo J., Chen C., Zhang B., Hu J. (2024). Recent Progress in Synthesis of MAX Phases and Oxidation & Corrosion Mechanism: A Review. Mater. Res. Lett..

[73] Hu W., Huang Z., Wang Y., Li X., Zhai H., Zhou Y., Chen L. (2021). Layered Ternary MAX Phases and Their MX Particulate Derivative Reinforced Metal Matrix Composite: A Review. J. Alloys Compd..

